# Individual Differences in Cue Weighting in Sentence Comprehension: An Evaluation Using Approximate Bayesian Computation

**DOI:** 10.1162/opmi_a_00052

**Published:** 2022-07-01

**Authors:** Himanshu Yadav, Dario Paape, Garrett Smith, Brian W. Dillon, Shravan Vasishth

**Affiliations:** Department of Linguistics, University of Potsdam, Germany; Department of Linguistics, University of Massachusetts, USA

**Keywords:** individual differences, interference effect, cue-based retrieval, approximate Bayesian computation, hierarchical modeling

## Abstract

Cue-based retrieval theories of sentence processing assume that syntactic dependencies are resolved through a content-addressable search process. An important recent claim is that in certain dependency types, the retrieval cues are weighted such that one cue dominates. This cue-weighting proposal aims to explain the observed average behavior, but here we show that there is systematic individual-level variation in cue weighting. Using the Lewis and Vasishth cue-based retrieval model, we estimated individual-level parameters for reading speed and cue weighting using 13 published datasets; hierarchical approximate Bayesian computation (ABC) was used to estimate the parameters. The modeling reveals a nuanced picture of cue weighting: we find support for the idea that some participants weight cues differentially, but not all participants do. Only fast readers tend to have the predicted higher weighting for structural cues, suggesting that reading proficiency (approximated here by reading speed) might be associated with cue weighting. A broader achievement of the work is to demonstrate how individual differences can be investigated in computational models of sentence processing without compromising the complexity of the model.

## INTRODUCTION

A well-established claim in sentence processing is that dependency completion—establishing who did what to whom—is driven by a cue-based retrieval process (Lewis & Vasishth, 2005; McElree, 2000; Van Dyke, 2007; Van Dyke & McElree, 2011). The key idea behind cue-based retrieval is that codependents like verbs and their associated subjects are identified and connected together via a content-addressable search in memory.

For example, consider the ungrammatical sentences in (1) below.

(1)  a. *The bodybuilder who worked with the trainers were complaining …
    b. *The bodybuilder who worked with the trainer were complaining …

Within the cue-based retrieval framework, the auxiliary verb *were* in (1) is assumed to initiate a search in memory for an appropriate subject noun phrase, using feature specifications such as plural (PL) and subject (SUBJ).^1^ In example (1a), one of the retrieval cues (SUBJ) matches with the subject, *bodybuilder*, which is the correct codependent of *were*. The other retrieval cue (PL), matches a distractor noun, *trainers*. A signature property of the retrieval framework is that a distractor noun can be probabilistically misretrieved due to such a partial feature match (Paape et al., 2021; Vasishth et al., 2019). These misretrievals have the effect that reading time at the auxiliary *were* is faster in (1a) compared to (1b), in which the distractor does not match any of the retrieval cues. The explanation for this so-called *facilitatory interference* effect is a race process resulting in statistical facilitation (Raab, 1962): In every trial, two processes are initiated simultaneously, each corresponding to one matching feature (SUBJ: *bodybuilder*, PL: *trainers*). Whichever process finishes first is the winner in that trial. Statistical facilitation refers to the fact that when the mean finishing times of the two processes are very similar, the mean reading time resulting from the race process will be faster than both of the mean finishing times corresponding to the individual processes.^2^ Facilitatory interference has been robustly observed in number-agreement configurations such as (1).

Similar effects have been shown in plausibility mismatch configurations (Cunnings & Sturt, 2018), negative polarity item licensing (Drenhaus et al., 2005; Vasishth et al., 2008; Xiang et al., 2009), and honorific processing (Kwon & Sturt, 2016). Given that cue-based retrieval parsing is intended as a comprehensive model of sentence processing, the phenomenon should generalize to any construction in which two partially matching nouns are available as retrieval candidates.

However, a construction that has been argued by Dillon et al. (2013) to be immune to facilitatory interference effects are antecedent-reflexive dependencies, as shown in the ungrammatical sentences in (2).

(2)  a. *The bodybuilder who worked with the trainers injured themselves …
    b. *The bodybuilder who worked with the trainer injured themselves …

**Figure 1.**
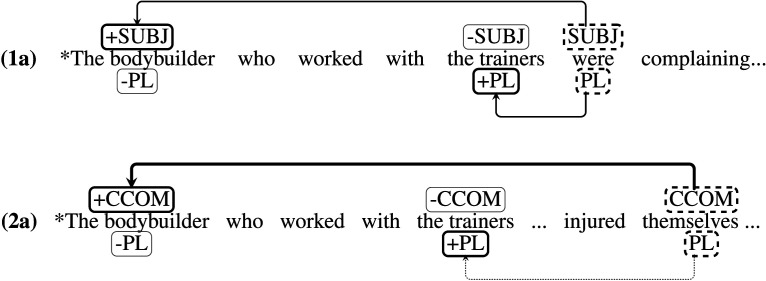
**The cue-weighting proposal: A 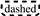 box represents a retrieval cue, a 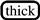 box represents a feature that matches a retrieval cue, and a 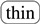 box represents a feature that does not match a retrieval cue.** In agreement dependencies, the cues are weighted equally, while in reflexive dependencies the c-command cue is assumed to be weighted more highly than the number cue, as indicated by the thickness of the lines.

Building on work by Sturt (2003), Dillon et al. (2013) argue that the search for an antecedent of a reflexive is guided exclusively by Principle A of the binding theory (Chomsky, 1981), which states that an anaphor must be bound by a c-commander within the same clause (see also Nicol & Swinney, 1989). The specific claim is that in examples like (2a) vs (2b), number marking on the reflexive (*himself* vs. *themselves*) is not used as a retrieval cue. This has the consequence that no difference in reading time is predicted at the reflexive in (2a) vs (2b). Other researchers (Cunnings & Sturt, 2014; Kush, 2013; Parker & Phillips, 2017) have proposed that although the c-command and plural retrieval cues are present at the reflexive, they are weighted differently in reflexives compared to subject-verb agreement: In reflexives, the weight of the structural cue versus the number cue is arguably higher, while in subject-verb agreement the cues have equal weights.^3^ The increased cue weighting reduces or eliminates the role of the plural cue, so that very little or no facilitatory interference occurs. This is shown schematically in Figure 1.

**Figure 2.**
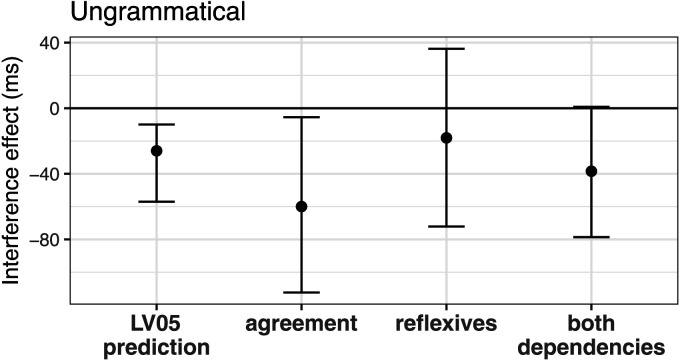
**The facilitatory interference effect in ungrammatical agreement vs. reflexive dependencies in the Dillon et al. (2013) data; also shown is the cue-based retrieval model’s predicted facilitatory interference effect for both dependency types (marked “LV05”).** The figure is reused with permission from Jäger et al. (2020).

Dillon et al. (2013) experimentally compared facilitatory interference effects in agreement and reflexive dependencies. They present data from 40 participants, which are summarized in Figure 2. Consistent with the cue-weighting account, we see a facilitatory interference effect in agreement dependencies, but no such effect in reflexive dependencies. The figure also shows the cue-based retrieval model’s quantitative predictions with equal weights for the retrieval cues across dependencies.

Consistent with the standard practice in psycholinguistics, the claim made by Dillon et al. and others that structure has a privileged role at retrieval in reflexives focuses on average behavior across all items and participants. However, focusing only on average behavior misses two potentially important details.

**Figure 3.**
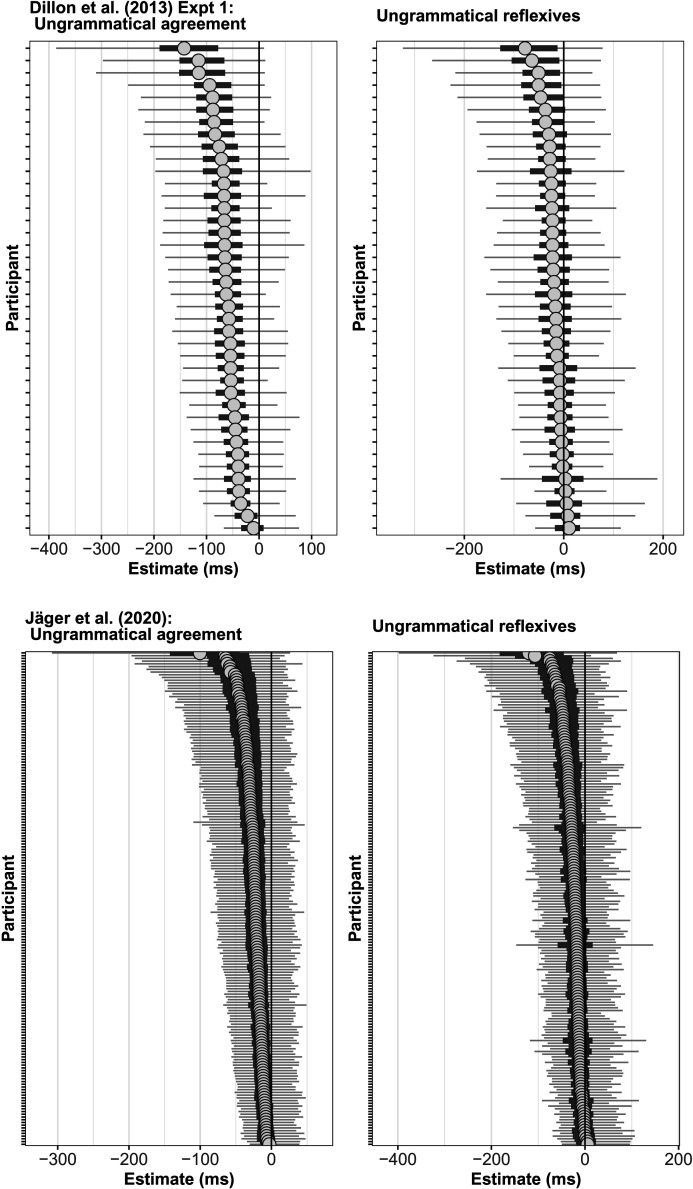
**Individual-level facilitatory interference effects in ungrammatical agreement vs. reflexive dependencies in the Dillon et al. (2013) and Jäger et al. (2020) data.** Shown are the shrunken estimates from a Bayesian hierarchical linear model fit to the data. The circle is the individual’s mean effect, the solid error bar is an 80% Bayesian credible interval, and the thinner line a 95% credible interval.

First, the 40 individual participants in the Dillon et al. study show differences in both dependency types in the magnitude of the facilitatory interference effect. Figure 3 (top) shows that in both reflexives and agreement dependencies, the magnitude of the estimated effect for individuals ranges from 0 ms to effects as large as −150 ms.^4^ This pattern of individual-level variability is replicable: In a large-scale replication attempt of the Dillon et al. (2013) experiment, Jäger et al. (2020) found a similar pattern in 181 new participants; see Figure 3 (bottom). Thus, while the population-level estimates are consistent with the cue-weighting theory, some of the individual-level effects for reflexives across studies are not consistent with it: There are people who do show facilitatory interference for reflexives.

**Figure 4.**
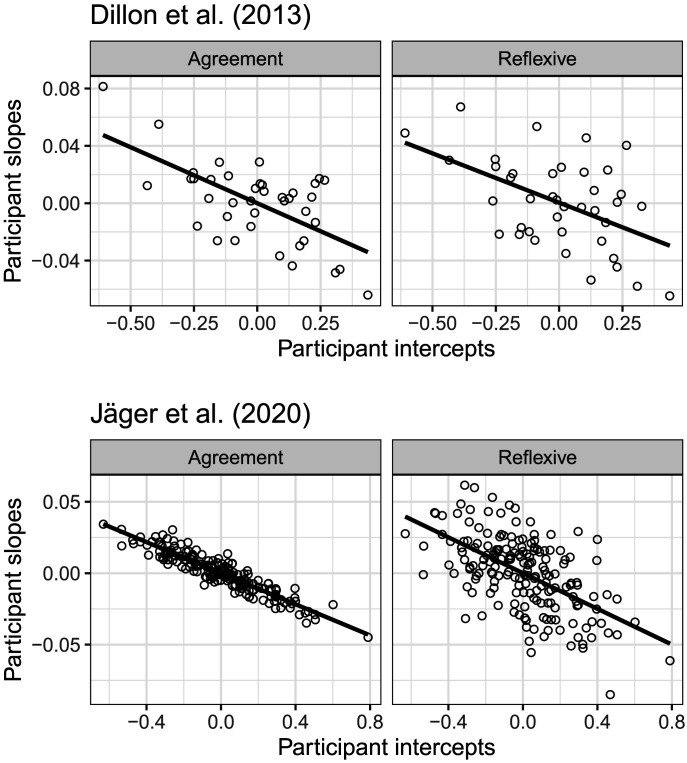
**The relationship between mean reading times and the magnitude of the interference effect in the Dillon et al. (2013) data and the Jäger et al. (2020) data.** Shown are the by-participant random intercept adjustments and the slope adjustments for the facilitatory interference effect. Both datasets show a clear pattern: slower participants show a larger magnitude of the facilitatory interference effect.

The second interesting aspect of the individual-level data is that the individual participants’ average reading speed^5^ is correlated with the magnitude of the facilitatory interference effect: the slower the participant, the larger the magnitude of the facilitatory interference effect. This correlation is displayed in Figure 4 for the Dillon et al. (2013) and the Jäger et al. (2020) datasets.

A simple explanation for this correlation would be that slower participants simply have higher standard deviations: a mean reading time with a relatively large value allows more room for variability around it than a mean reading time that has a small magnitude. There is, however, an alternative, theoretically grounded explanation for the observed variability as well as for the correlation with reading times: Individuals might differ in their cue weighting, and comparatively lower weight for the structural cue could be associated with slower reading speed, making less-fluent readers more susceptible to facilitatory interference.^6^

Under these assumptions, participants with higher cue weighting for the structural (c-command or subject) cue should show no facilitatory interference, while participants with approximately equal cue weighting should show facilitation.

There is evidence for individual differences in cue weighting in the literature: memory research suggests that some individuals learn to use certain cues more effectively and reliably compared to other cues (Danker et al., 2011). Sohn et al. (2004) have shown that different training procedures lead participants to weight retrieval cues differently in a recall task. In sentence processing, some individuals could learn to use structural cues more effectively and reliably compared to nonstructural cues, and these individuals may also be more fluent readers. This view receives some support from the study reported by Traxler et al. (2012) on relative clauses: Traxler et al. found that readers with slower mean reading times experienced more processing difficulty in object relative clauses with misleading semantic cues (*The director that the movie pleased* …) than readers with faster mean reading times. Traxler et al. hypothesized that due to fast readers’ greater experience with the object-relative structure, the detrimental effect of the misleading semantic cue was reduced in fast readers.

Assuming that fast readers presumably also have more experience with other linguistic structures such as reflexives, their susceptibility to interference from structurally unavailable distractor nouns may be reduced in a similar manner.

Our proposed connection between language experience and cue weighting would also be consistent with results from nonnative speakers, whose reading speed has been found to correlate with their proficiency (Roberts & Felser, 2011), and who have been argued to assign higher weights to discourse-based cues over structural cues (Cunnings, 2017). In children, reading speed is positively correlated with text-level comprehension (Cutting & Scarborough, 2006; Jenkins et al., 2003), and among adults, highly skilled readers have been found to have shorter average fixation durations in eye-tracking than less-skilled readers (Underwood et al., 1990). Furthermore, reading speed as a measure has high reliability (Cunnings & Fujita, 2020; James et al., 2018), suggesting that it captures stable underlying differences between individuals.

Given the possibility of differences in cue weighting among individuals, the claim by Dillon et al. (2013) and others could be reformulated as follows: in reflexives, the majority of the individual participants should have higher cue weighting for the c-command cue than for the plurality cue. By contrast, in subject-verb number agreement, the majority of participants should exhibit equal cue weighting for the subject and plurality cues. Furthermore, if faster, more-skilled readers are less vulnerable to facilitatory interference, faster reading speed should correlate with higher weight for the structural cue over the plurality cue.

These predictions about individual-level variability in cue weighting and reading speed can be investigated quantitatively in the Lewis and Vasishth (2005) cue-based retrieval model. The predictions can be evaluated by (a) estimating, within the cue-based retrieval model, cue weighting and reading speed parameters separately for each individual participant, (b) deriving the predicted reading times from the model for each participant, and then (c) comparing these predicted reading times to the observed reading times for individuals. Stable alignment between the predicted and observed values would suggest that the model adequately captures the assumed underlying processes. Furthermore, given the observed correlation between reading speed and facilitatory interference in the data, a correlation between the model parameters that encode reading speed and cue weighting is expected.

We discuss the details of the cue-based retrieval model and the relevant parameters next.

### The Lewis and Vasishth (2005) Model

The Lewis and Vasishth (2005) cue-based retrieval model, abbreviated below as LV05, is based on Adaptive Control of Thought—Rational (ACT-R) (Anderson & Lebiere, 2014) and assumes that during retrieval, activation spreads to the memory chunks that match the retrieval cue. The more cues are matched by a chunk, the more activation it receives.

Simplifying slightly (cf. Engelmann et al., 2019), the total activation of a memory chunk *i* is given by

$$A_{i}=B_{i}+\sum_{j}W_{j}S_{ji}+\epsilon_{i}$$ (1)

where *B*_*i*_ is the baseline activation of the chunk *i* determined by past retrievals. A memory chunk *i* receives spreading activation from all matching cues *j* depending on the associative strength *S*_*ji*_ between the cue *j* and the chunk *i*, and the cue’s weight *W*_*j*_. The amount of activation spread determines the total activation of the memory chunks.

ϵ_*i*_ is Gaussian noise added to activation of the chunk *i*, such that ϵ_*i*_ ∼ *Normal*(0, *σ*); *σ* represents the standard deviation of the normal. The fact that the activation values are noisy is crucial to the facilitatory interference effect: Even when two chunks receive the same amount of activation from the retrieval cues—such as *bodybuilder* and *trainers* in (1a)—the final activation values and the associated latencies can differ from trial to trial due to noise.

In a particular trial, the memory chunk that happens to have the highest activation is retrieved.^7^ The time taken to complete the retrieval is determined by the activation level of the retrieved item: when activation is higher, the retrieval is faster. The retrieval time, *RT*_*i*_, of a chunk is a negative exponential function of its activation at the time of retrieval:

$${RT}_{i}={Fe}^{\left(-{fA}_{i}\right)}.$$ (2)

*F* and *f* are two scaling parameters—the latency factor and the latency exponent, respectively. Latency factor and latency exponent reflect “surface-level” processing independent of the activation level of the chunks and, unlike the activation level, do not vary between trials. The latency factor represents the *general reading speed* of an individual and may, inter alia, include lexical access time, encoding time and motor response time.

**Figure 5.**
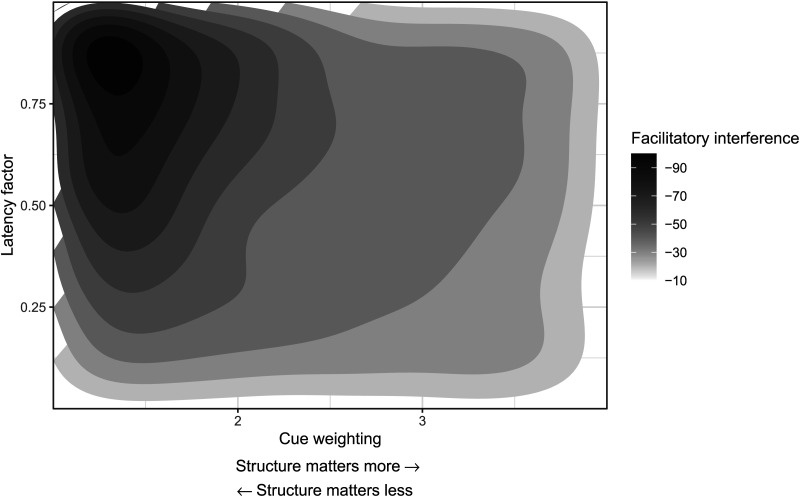
**The facilitatory interference effect (in milliseconds) predicted by the model as a function of latency factor and cue weighting.** The gradient from the light to darker shade represents the increasing magnitude of the facilitatory interference effect for the given values of cue weighting and latency factor. Cue weighting is the ratio of the weights of structural and nonstructural cues. The facilitatory effect increases linearly with latency factor when cue weighting is small; the effect decreases exponentially with increase in cue weighting.

In multiple-match scenarios, as in (1a), the activation distributions—and therefore the latency distributions—of the two candidate chunks have a larger overlap compared to (1b). The larger the overlap, the larger the observed speedup due to statistical facilitation. Since retrieval time of a chunk is a function of cue weights, the amount of facilitatory interference is modulated by the relative weights of the retrieval cues; see Figure 5. If each candidate chunk matches one retrieval cue and the cues have equal weights, the spreading activation for both chunks is the same. By contrast, if the structural cue has higher weight, the target chunk receives more activation, leading to reduced overlap and less facilitation. To understand the inner workings of the model in detail, see Lewis and Vasishth (2005) and Engelmann et al. (2019).

A convenient way to operationalize cue weighting is as the ratio of the weights of structural versus the nonstructural cue used in the retrieval (Engelmann et al., 2019). Cue weighting larger than 1 means more weight is given to the structural *subject* cue over the nonstructural *number* cue.^8^

For the present purposes, we are interested in estimating the parameters *W*_*j*_, that is, the weights of the c-command and number cues, expressed as a ratio, and *F*, that is, the latency factor, for each individual participant. It is plausible to assume that the latency factor is the main source of variability in average reading speed between participants: The word-level processing of more experienced, fluent readers can be expected to be faster and more automatic (e.g., Joo et al., 2021; Kuperman & Van Dyke, 2011; Logan, 1997; Samuels & Flor, 1997), which should lead to faster reading irrespective of the memory processes triggered during the completion of dependencies. For the present purpose, we will use reading speed as a proxy for language experience.^9^

Our hypothesis is that fast word-level processing as indexed by small values of *F* tends to coincide with high values for the *W*_*j*_ parameter that controls the weight of structural over nonstructural cues used at the sentence level, and that language experience is the connecting factor. Participants who read fluently and automatically should therefore be less susceptible to interference from structurally illicit distractors, and there should thus be a negative correlation between latency factor and cue weighting.

### Robust Estimation of Individual Differences Using Approximate Bayesian Computation

Parameter estimation is a key challenge in modeling individual differences. Suppose that we collect some data, *y*, from participants in an experiment, and we assume that the data *y* come from a function of a vector of parameters θ: *y* ∼ *f*(θ). The statement *y* ∼ *f*(θ) represents a model where θ = {*θ*_1_, *θ*_2_, …, *θ*_*m*_}. The aim of parameter estimation is to compute the join posterior distributions of the parameters θ that would have generated the observed data *y*.

Parameter estimation for the LV05 model is difficult because the model is nondeterministic and its likelihood cannot easily be expressed analytically, unless we drastically simplify the model (e.g., in Lissón et al., 2021; Nicenboim & Vasishth, 2018; Paape et al., 2020). Therefore, we cannot employ the standard Bayesian estimation method for estimating parameters of the LV05 model.

In the published modeling work on cue-based retrieval, grid search is the standard approach used to estimate the parameters in the model (Engelmann et al., 2019; Mätzig et al., 2018). In grid search, the parameter space for each parameter *θ*_*i*_ is divided into *n* equally spaced points, {*v*_*i*,1_, …, *v*_*i*,*n*_} and all possible combinations are taken to create *n*^*m*^ grid points. Each grid point is evaluated by generating a model prediction, and a grid point that provides the best fit to the observed data is chosen as the estimated parameter value (or set of values, in case multiple parameter values provide the best fit).

There are several important limitations to the grid search method (Kangasrääsiö et al., 2019). First, grid search is a brute-force method, and therefore inefficient; the number of computations increases exponentially as the number of parameters *m* increases. It is therefore not computationally feasible to compute individual-level as well as population-level estimates using this approach. Second, grid search usually delivers a point estimate of the parameter value; uncertainty about the parameter estimates cannot be computed.

As Kangasrääsiö et al. (2019) point out, a better way to estimate model parameters is Bayesian estimation. This approach allows us to estimate the posterior distributions of the parameter values given the observed data, that is, *π*(*θ*|*y*) using Bayes’s rule, which is shown in Equation 3.

$$\pi \left(\theta |y\right)=\frac{\pi \left(y|\theta \right)\pi \left(\theta \right)}{\pi \left(y\right)}$$ (3)

As Bayes’s rule states, the estimation of the posterior distribution, *π*(*θ*|*y*) requires knowledge about the likelihood, *π*(*y*|*θ*) (i.e., probability density of seeing the data for given parameter value) and the prior knowledge about *θ* before *y* is available, that is, *π*(*θ*). However, for the LV05 model, the likelihood function, that is, *π*(*y*|*θ*) is difficult to express mathematically.

For such situations, approximate Bayesian computation (ABC) has emerged as an effective tool for approximating the posterior distributions of the parameters (Palestro et al., 2018; Sisson et al., 2018). ABC uses an approximation of the likelihood function to estimate the posterior distribution for the parameters. Even when the likelihood function is unknown, the model can still generate simulated data for given parameter values. The ABC method consists of comparing the observed data with the simulated data. Since ABC is rooted in Bayesian estimation techniques, it also computes the uncertainty bounds on the parameter estimates.

A simple ABC sampler works as follows:

1. Draw a proposal θ* from the prior distribution, that is, θ* ∼ *π*(*θ*).
2. Simulate data *x* from the model conditional on θ*.
3. If the simulated data *x* is “close” to the observed data *y*, accept θ* as a sample for the posterior distribution. The “closeness” between simulated data *x* and observed data *y*, that is, *dist*(*x*, *y*) approximates the likelihood for θ*.

If the above steps are carried out repeatedly, we can obtain samples from the approximate posterior distribution.

One approach to approximating the likelihood is to weight the proposal θ* based on distance between simulated and observed data, *dist*(*x*, *y*). If the simulated data *x* corresponding to a proposed value θ* is closer to the observed data *y*, the proposed θ* will have higher weight. The weights can be assigned using a distribution centered at zero, such that if *dist*(*x*, *y*) is zero, the proposed θ* will have highest weight, and as *dist*(*x*, *y*) increases, weight decreases. ABC uses summary statistics *S*(·)—such as the mean—of the observed and the simulated data to compute *dist*(*x*, *y*). The weight assigning function is called a kernel function. Suppose that the kernel function is Ψ(*dist*(*x*, *y*)|*δ*).

Here, *δ* is the tolerance parameter, which determines the degree of the approximation. The lower the value of *δ*, the better the approximation. When *δ* approaches 0, the approximation becomes exact:

$$\pi \left(\theta |y\right)\propto \int_{X}\Psi \left(\text{dist}\left(S\left(x\right),S\left(y\right)\right)|\delta \right)\pi \left(x|\theta \right)\pi \left(\theta \right)dx,$$ (4)

where *X* is the support of the simulated data from the model.

The intractable likelihood term *π*(*x*|*θ*) cancels out while calculating the probability of accepting a proposal in posterior simulation algorithms.

We use the ABC method to estimate the participant-level and population-level parameters of the cue-based retrieval model. Our parameter estimation problem is more complex than the simple ABC algorithm presented above: in order to model individual differences, we need to estimate both individual- and population-level parameters simultaneously. For such a situation, Turner and Van Zandt (2014) propose a hierarchical Gibbs ABC algorithm. The details of this method are explained later, but in essence, the idea is to first draw samples for the individual-level parameters using ABC and then sample for each population-level parameter from a distribution conditioned on all other parameters.

Returning to our main research question, we now describe a computational evaluation of the two claims that follow from the cue-weighting proposal: (i) in reflexives, most individuals should have higher cue weighting for the c-command cue compared to the plurality cue and (ii) in agreement, most individuals should have equal cue weighting for the subject and plurality cues. Furthermore, we investigate (iii) whether there is a correlation between cue weighting and reading speed.

## MODELING INDIVIDUAL DIFFERENCES IN THE CUE-BASED RETRIEVAL MODEL

We obtained 13 datasets from four published studies (Dillon et al., 2013; Jäger et al., 2020; Lago et al., 2015; Wagers et al., 2009) that report the facilitatory interference effect. Table 1 lists the datasets along with number of participants in the experiment and population-level mean facilitatory interference effect. Out of these 13 datasets, 11 tested subject-verb agreement dependencies (Dillon et al., 2013; Lago et al., 2015; Wagers et al., 2009), and the remaining two datasets (Dillon et al., 2013; Jäger et al., 2020) investigated both subject-verb agreement and reflexive dependencies.

**Table 1.** Facilitatory interference effect data used for estimating parameters of the LV05 model.

**Dataset**	**Number of Participants**	**Interference Effect (in milliseconds)**
**Subject-verb agreement dependency**		
Dillon et al. (2013) Exp 1	40	−60 [−111, −11]
Jäger et al. (2020)	181	−27 [−47, 2]
Lago et al. (2015) Exp 1	32	−27 [−55, 0]
Lago et al. (2015) Exp 2	32	−23 [−54, 7]
Lago et al. (2015) Exp 3a	32	−13 [−29, 2]
Lago et al. (2015) Exp 3b	32	−13 [−33, 6]
Wagers et al. (2009) Exp 2	28	−23 [−49, 1]
Wagers et al. (2009) Exp 3 (Singular subject)	60	−18 [−46, 9]
Wagers et al. (2009) Exp 3 (Plural subject)	60	−4 [−39, 30]
Wagers et al. (2009) Exp 4	44	−28 [−53, −3]
Wagers et al. (2009) Exp 5	60	−20 [−44, −4]
**Antecedent-reflexive dependency**		
Dillon et al. (2013) Exp 1	40	−18 [−73, 36]
Jäger et al. (2020)	181	−23 [−49, 2]

*Note*. The table lists the published datasets along with the number of participants and population-level mean interference effect. The square brackets show 95% credible intervals around the population-level interference effects, that is, there is a 95% probability that the value of the population-level interference effect lies within this range, given the statistical model and data.

Using these datasets, we implemented hierarchical ABC to estimate the participant-level and population-level parameters. The code and data are available from https://osf.io/3na9q/. The latency factor (which modulates retrieval time) and cue weighting were estimated simultaneously for each participant; the computational details behind the ABC algorithm are explained in Appendix S1 in the Supplemental Materials.

For each dataset, we fit a Bayesian hierarchical model with varying intercepts and slopes for participants and items, where reading time is the dependent variable and condition (multiple-match vs. single-match) is a sum-coded predictor (Schad et al., 2020). As discussed earlier, each fitted model provided shrunken estimates of individual-level facilitatory interference effects. We used these individual-level shrunken estimates as data for the cue-based retrieval model.

As we describe now, the hierarchical ABC method provided estimates of participant-level latency factor and cue weighting, population-level mean latency factor and cue weighting, population standard deviation for latency factor and cue weighting, and the correlation between latency factor and cue weighting.

Suppose that *y*_*j*_ are the data (interference effect) associated with the *j*^*th*^ participant. *LF*_*j*_ and *CW*_*j*_ are the latency factor and the cue weighting parameters, respectively, for the *j*^*th*^ participant. We assume that the human data *y*_*j*_ are generated by the LV05 model with the parameters *LF*_*j*_ and *CW*_*j*_:

$$y_{j}∼\text{Model}\left({LF}_{j},{CW}_{j}\right).$$ (5)

We further assume that *LF*_*j*_ and *CW*_*j*_ come from a bivariate normal distribution with population means *μ*_*LF*_ and *μ*_*CW*_, standard deviations *σ*_*LF*_ and *σ*_*CW*_, and correlation parameter, *ρ*:

$$\left(\begin{array}{c} {LF}_{j}\\ {CW}_{j} \end{array}\right)∼{𝒩}_{2}\left(\left(\begin{array}{c} \mu_{LF}\\ \mu_{CW} \end{array}\right),\left(\begin{array}{cc} \sigma_{LF}^{2} & {\rho \sigma }_{LF}\sigma_{CW}\\ {\rho \sigma }_{LF}\sigma_{CW} & \sigma_{CW}^{2} \end{array}\right)\right).$$ (6)

The goal here is to obtain posterior estimates of the individual-level parameters *LF*_*j*_ and *CW*_*j*_ and the population-level parameters *μ*_*LF*_, *μ*_*CW*_, *σ*_*LF*_, *σ*_*CW*_, and *ρ*. Assuming that the data come from *n* participants, we have 2*n* + 5 parameters to estimate, that is, *LF*_*j*_, *CW*_*j*_ for each participant and five population-level parameters *μ*_*LF*_, *μ*_*CW*_, *σ*_*LF*_, *σ*_*CW*_, and *ρ*.

### Results

#### Cue Weighting in Agreement and Reflexive Dependencies.

**Figure 6.**
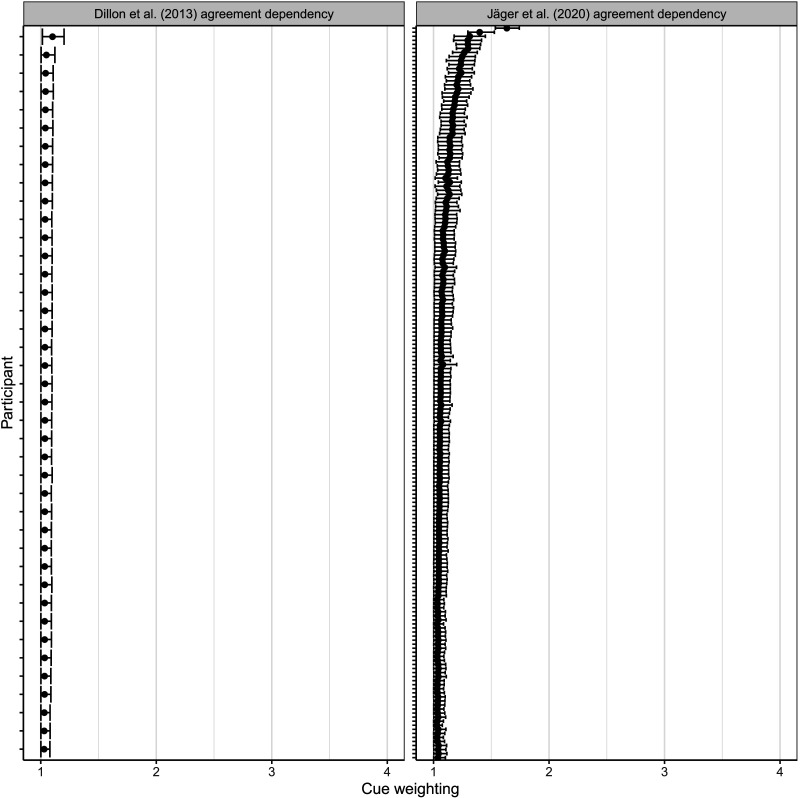
**Participant-level cue weighting for agreement dependency data in Dillon et al. (2013) and Jäger et al. (2020).** Shown are the 95% credible intervals for each individual’s estimate, along with the estimated mean parameter value for each participant.

**Figure 7.**
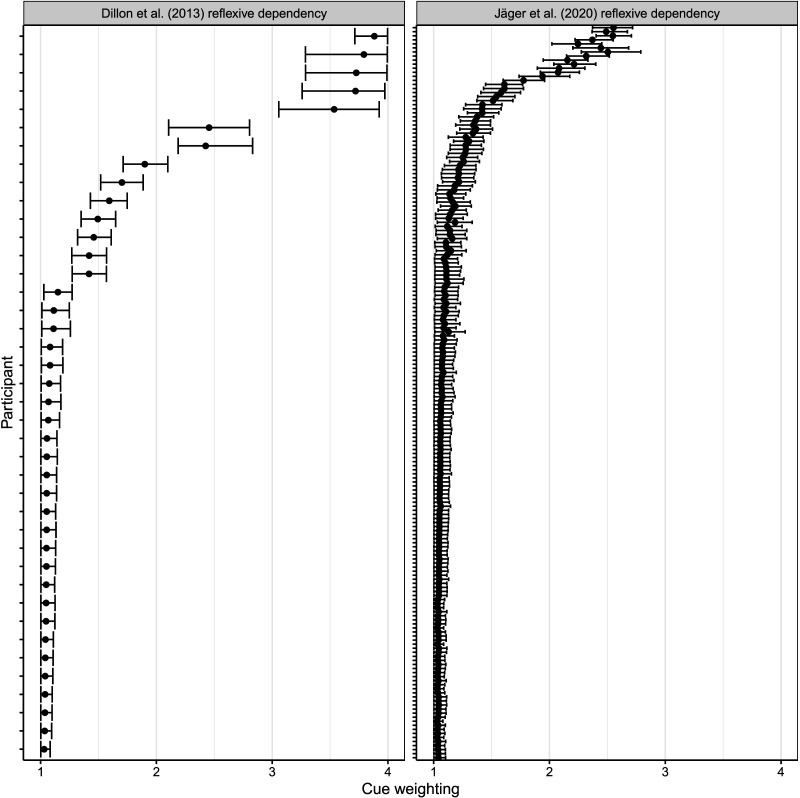
**Participant-level cue weighting for reflexive dependency data in Dillon et al. (2013) and Jäger et al. (2020).** Shown are the 95% credible intervals for each individual’s estimate, along with the estimated mean parameter value for each participant.

First, we focus on the Dillon et al. (2013) and Jäger et al. (2020) experiments. Figures 6 and 7 compare the distribution of participant-level cue weighting in agreement and reflexive dependencies for these two studies.

The individual-level estimates for agreement show that in the Dillon et al. (2013) and Jäger et al. (2020) data, almost all participants have equal cue weighting, that is, the weight ratio between the structural cue and the number cue is close to 1:1, consistent with the cue-weighting account. For reflexives, the two experiments show similar patterns: About one quarter of participants in the original Dillon et al. (2013) have estimates for cue weighting close to or higher than 2:1 in favor of the structural cue, which is consistent with the cue-weighting account. At the same time, many participants have cue weighting close to 1:1. The reflexives data from Jäger et al.’s (2020) replication study also have a minority of participants with high cue weighting, but there are also many participants with cue weighting estimates close to 1:1. The overall pattern in the replication study is noticeably more graded, but this may be due to the larger sample size.

**Figure 8.**
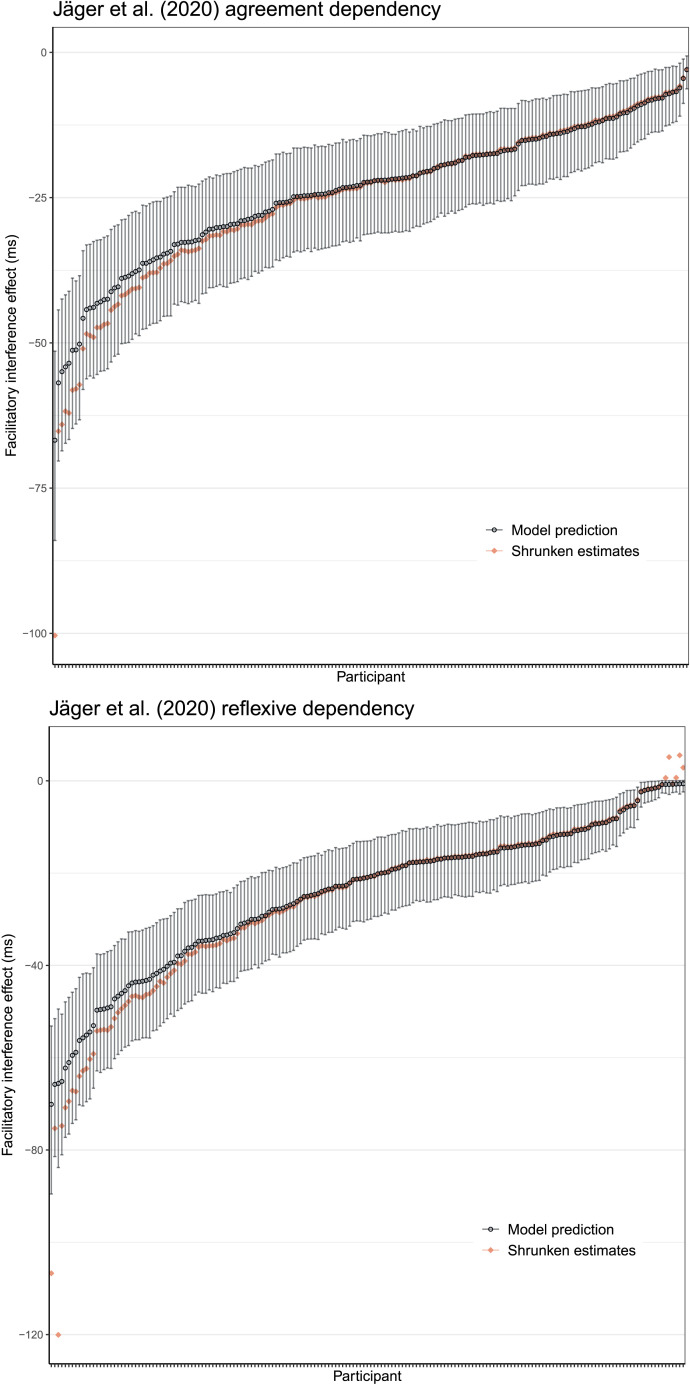
**Posterior predicted interference effects for individuals in agreement and reflexive dependencies, derived from the cue-based retrieval model after estimating individual-level parameters for cue weighting.** Shown are the posterior mean and 95% credible interval, along with the estimate of the shrunken mean for each participant.

For the Jäger et al. (2020) study, simulating data based on the individual participants’ parameter estimates for cue weighting and latency factor and computing the facilitatory interference effect yields values very close to the shrunken estimates based on the original data. Figure 8 shows the model estimates alongside the shrunken estimates from the hierarchical linear models. Although not shown here, the model can also capture the individual-level estimates from the Dillon et al. (2013) data (see Appendix).

#### Correlation Between Cue Weighting and Reading Speed.

**Figure 9.**
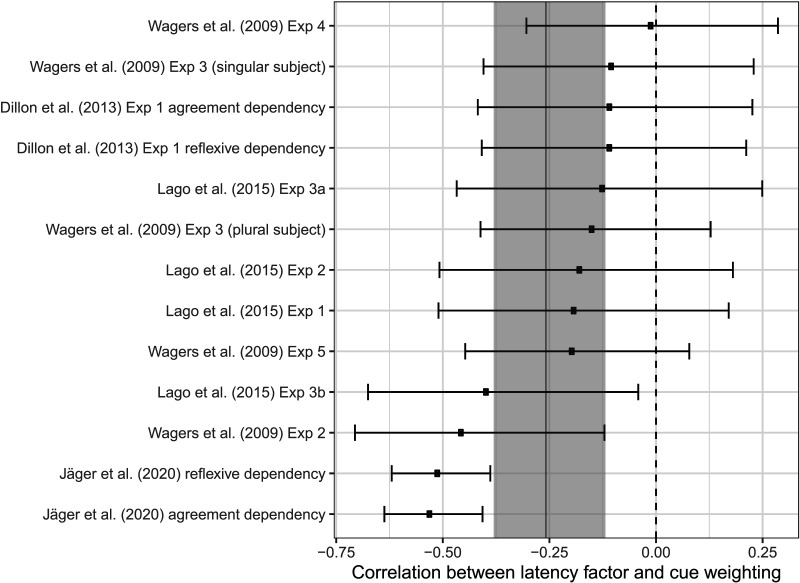
**Estimated correlations between participant-level latency factor and cue weighting for 13 datasets that investigated the facilitatory interference effect.** Shown are the posterior distributions of the correlation parameters (mean and 95% credible interval); the dark band is the posterior distribution of the overall correlation, derived from a random-effects meta-analysis.

We now analyze each of the 13 datasets separately to investigate the relationship between cue weighting and latency factor. Figure 9 shows the posterior distribution of the correlation between cue weighting and reading speed, that is, the latency factor, across the 13 datasets. Most of the estimates are negative, in line with the prediction that slower, less-skilled readers who have high latency factors should have low cue weighting, that is, weight ratios close to 1:1. In order to synthesize the evidence relating to the correlation from all the available studies, we conducted a random-effects meta-analysis. We use a Bayesian meta-analysis method that is based on the Fisher z-transformation of correlation estimates (Fisher, 1921; Zhang et al., 2017). The meta-analysis model is described in Appendix S2 in the Supplemental Materials.

The meta-analytical estimate for the correlation parameter is −0.26, and the associated 95% credible interval is [−0.38, −0.12], as shown by the shaded region in Figure 9. Taken together, the data thus indicate a negative correlation between cue weighting and latency factor, consistent with the idea that slower readers tend to have equal cue weighting, and that the faster the participant, the higher the weighting in favor of the structural cue.

### Discussion

Based on the two studies that investigated both subject-verb agreement and reflexive dependencies, Dillon et al. (2013) and Jäger et al. (2020), we compared individual-level cue weighting for each of the dependencies. Results for agreement dependencies showed equal cue weighting for the majority of participants in both experiments, consistent with the cue-weighting account. By contrast, the results for reflexive dependencies showed that both the Dillon et al. (2013) and Jäger et al. (2020) data do have some participants with higher cue weighting for the structural cue, but the majority of the participants do not have higher cue weighting.

It is interesting to note here that, based on the average estimates from their respective datasets, Dillon et al. (2013) and Jäger et al. (2020) came to different conclusions. Dillon et al. concluded that reflexives are processed differently from subject-verb agreement, in that number features are effectively ignored during retrieval due to Principle A. In contrast, Jäger et al. concluded, again based on average estimates, that their larger dataset had showed no indication of a difference in processing between agreement and reflexive dependencies.

The individual-level estimates qualify both conclusions: in both the Dillon et al. (2013) and the Jäger et al. (2020) data, there are in fact participants that mostly ignore the number features of structurally inaccessible noun phrases in reflexive dependencies, as indicated by higher weighting of the structural cue. On the other hand, a majority of participants show processing profiles for reflexives that are similar to agreement dependencies, where the number cue of the distractor matters.

The present findings are thus inconsistent with the strongest possible version of the cue-weighting proposal, which is that *all* readers should show high cue weighting for reflexives. They are also inconsistent with the next-strongest version of the proposal, which is that *most* readers should show high cue weighting for reflexives. Instead, our findings show that there is only a nonnegligible minority group of readers in the two studies who show high cue weighting for reflexives.

The meta-analysis of our simulations based on all 13 available datasets shows that cue weighting in favor of the structural cue correlates with reading speed, such that faster readers are more likely to assign higher weight to the structural cue. The correlation estimates vary between studies, and the credible intervals of the correlation parameter cross zero in many cases, especially for studies with much fewer participants than the higher-powered study of Jäger et al. (2020). Nevertheless, the evidence as a whole, as quantified by the meta-analysis, is compatible with the notion that faster, more skilled readers assign more weight to structural cues during sentence processing. While most of the studies used in our simulation tested subject-verb agreement dependencies, this relationship also holds for reflexive dependencies, as shown by the estimate for the Jäger et al. and Dillon et al. (2013) studies (see Figure 9).

## GENERAL DISCUSSION

The present article addressed two questions related to individual differences in sentence comprehension. The first question was whether retrieval cues are weighted differently in subject-verb agreement and reflexive agreement configurations. The second question was whether participants who read more quickly on average also show larger facilitatory interference effects. In order to answer these questions, we used approximate Bayesian computation to fit individual- and population-level parameters of the cue-based retrieval model of Lewis and Vasishth (2005) to 13 datasets. ABC allows us to use well-motivated but complex models of sentence processing to directly test our questions using experimental data. We now discuss the results for the cue weighting and correlation questions in turn.

### Cue Weighting in Agreement and Reflexive Dependencies

**Figure 10.**
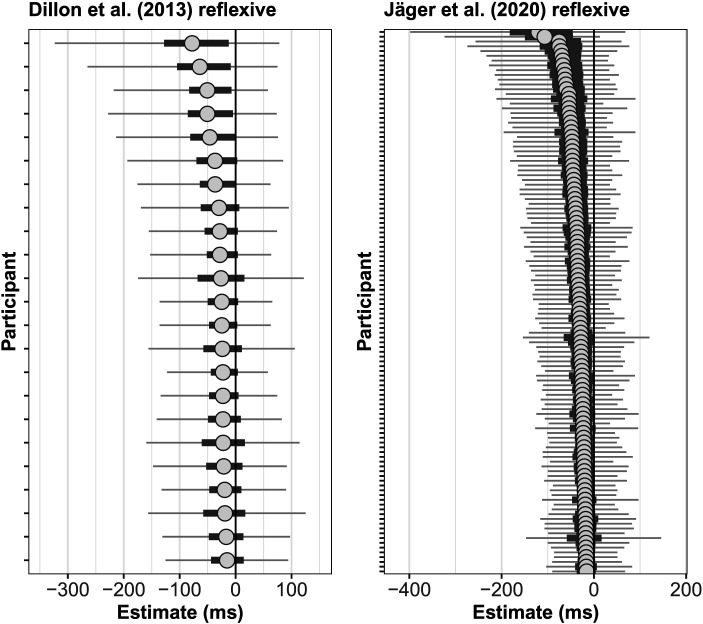
The facilitatory interference effect associated with the participants who had approximately equal cue weights for c-command and number cue in reflexive dependencies.

For the studies that provided data on reflexive dependencies (Dillon et al., 2013; Jäger et al., 2020), we found that a minority of participants did have higher cue weighting for the structural cue than the nonstructural cue in these dependencies, but also that most participants did not. This suggests that some participants do indeed strongly adhere to Principle A during online sentence comprehension. This prevents misretrievals of a distractor word and thus blocks the facilitatory interference effect that is often observed in number agreement contexts. However, for the majority of participants, there is no indication of a difference in processing between agreement and reflexives. For participants who weigh structural and nonstructural cues approximately equally, facilitatory interference occurs in both constructions (see Figure 10). Overall, the results suggest that the cue-weighting hypothesis only holds for a subset of English native speakers.

The subset of participants who have higher weighting for the structural cue show weak or absent facilitatory interference, that is, a magnitude of smaller than 8 ms. The estimated value of cue weighting for these participants has a logarithmic relationship with facilitatory interference: the smaller the magnitude of the facilitatory interference effect, the higher the weighting for the structural cue over the nonstructural cue. By contrast, participants with facilitatory interference effects of a magnitude over 8 ms have equal cue weighting for structural and nonstructural cues.

For the subset of participants who do weight the structural cue more highly, it is reasonable to ask what the cause of the different weighting is. Two possible reasons are as follows:

1. There are differences in the “level of representation” accessed by each dependency (Dillon et al., 2013). The reflexive dependencies are possibly keyed to more semantic/notional number than are agreement dependencies, which causes reflexives to be less sensitive to morphosyntactic number and hence to have higher weight for the structural cue. This claim has independent support in Kreiner et al. (2013), who found that in processing reflexive dependencies where the antecedent and reflexive matched in notional number, the mismatch in morphosyntactic number between the reflexive and the antecedent noun did not incur a processing cost. By contrast, subject-verb agreement showed a reliable mismatch cost due to morphosyntactic number even when the subject noun and the verb had the same notional number.
2. Reflexives and agreement dependencies differ in predictability. In antecedent-reflexives constructions, the reflexive is generally not expected given the preceding context. Hence, unlike agreement dependencies, there is no prediction for an upcoming co-dependent that could facilitate dependency completion. A possible implication is that comprehenders adopt a more conservative approach to resolving reflexive dependencies. This could change the priority given to different cues in the retrieval strategy used to resolve a reflexive, by giving more priority to the more diagnostic structural cues (see also Parker & Phillips, 2017).

However, the above possibilities are underspecified and untested in the context of the cue-based retrieval model, which we used in this study. In order to verify them, future work with cue-based retrieval theories will need to formalize how linguistic cues get learned over time for different dependencies.

A broader conclusion to be drawn from our simulations is that a focus on modeling population-level effects may mask theoretically interesting variation at the individual level. While it has become the norm in psycholinguistics to include random intercepts and slopes by participant and by item to guard against anticonservative conclusions at the population level (Barr et al., 2013), the magnitude and shape of the observed interindividual variation is seldom discussed. In principle, any two linguistic constructions may be distinguished both by the amount of possible variation between individuals, as has been claimed for subject-verb agreement versus reflexive dependencies, but also by whether differences between speakers are more quantitative or more qualitative in nature (Navarro et al., 2006; Rouder & Haaf, 2021). With regard to the weighting of structural cues in sentence processing, our results suggest that there is more variability between speakers for reflexive dependencies than for agreement dependencies. However, whether it is possible to experimentally identify a clearly delimitable subgroup of speakers who show strong adherence to Principle A during the processing of reflexive dependencies is not yet clear.

What underlying factors could plausibly distinguish readers with high cue weighting from readers with low cue weighting? One candidate factor we suggest is language experience, as indexed by reading speed (see discussion below). Nevertheless, it is an open question whether readers with a high weighting of the structural cue generally have a more strongly developed or less “gradient” grammar compared to those with low cue weighting. Rather than having a general preference for structural cues, it is also possible that they treat Principle A in particular as a “hard” constraint, whereas other participants may treat it as more of a “soft” constraint (Sorace & Keller, 2005). This latter view seems to be more consistent with the data, given that structural cues are also used in the resolution of agreement dependencies, for which our datasets show no participants with high cue weighting estimates.

### The Relationship Between Latency Factor and Cue Weighting

We turn now to the relationship between reading speed and cue weighting. The possibility of a population-level correlation between latency factor and cue weighting is suggested by the empirical data of Dillon et al. (2013) and Jäger et al. (2020), as well as by the hypothesis that more skilled readers read more quickly and might apply syntactic constraints more strictly. We evaluated this question using all 13 datasets available. After fitting the cue-based retrieval model using ABC, a meta-analysis of the population-level correlations between the latency factor (a parameter indexing average reading speed in the model) and cue weighting showed a correlation of −0.26 (95% CrI [−0.38, −0.12]): The faster participants read, the higher they tend to weight the structural cue over the number cue. This preliminary result should be tested in a new, confirmatory experiment; however, it is suggestive first evidence for a relationship between reading speed as an index of reading skill and how strongly participants adhere to grammatical constraints during reading.

### Limitations of the Present Study, and Future Directions

Our method provides new ways of investigating individual differences in sentence comprehension; however, there are a number of limitations that should be addressed in follow-up studies.

First, we have focused on facilitatory interference effects in ungrammatical sentences in this article. The main reason behind this decision is that the evidence for facilitatory interference in ungrammatical sentences—that is, distractor-induced speedups—in the literature is relatively robust. By contrast, the evidence for inhibitory interference in grammatical sentences—that is, distractor-induced slowdowns—which is also predicted by the Lewis and Vasishth (2005) model, is much more mixed (Jäger et al., 2017). While it is important to understand how individual-level latency factors, cue weightings, and their correlation behave in grammatical sentences (e.g., *The bodybuilder who worked with the trainer[s] was complaining* … ), the LV05 model likely needs to be augmented to account for the full range of results. Work is underway to apply an augmented version of the LV05 model to both grammatical and ungrammatical configurations simultaneously to gain a more complete picture of the individual variability in agreement and reflexive constructions. Nevertheless, it is still worthwhile to investigate how well the base LV05 model captures participants’ behavior across the different dependencies in ungrammatical sentences, as we have done in the present work.

An additional limitation comes from the fact that we used the individual-level estimates of the facilitatory interference effect from the published data as the basis for our simulations. These estimates come with a high degree of uncertainty. That is not an issue specific to the data we used: reading times are highly variable and studies are often underpowered (see appendix B of Jäger et al., 2017). The high uncertainty of the data increases the uncertainty of our simulation-based parameter estimates. One solution to this problem is to collect more data from each participant. This provides more accurate estimates of individual parameter values, even though longer experiments may result in adaptation to the linguistic manipulation and reduced differences between conditions (Fine et al., 2013). Finding the right balance here is a challenging task for future work.

Our implementation of the Lewis and Vasishth (2005) model assumes that individual-level parameter values are sampled from a unimodal Gaussian distribution that is centered around the population-level mean. This assumption constrains the allowed variability across individuals, which is assumed to be quantitative rather than qualitative in nature (Haaf & Rouder, 2019; Navarro et al., 2006). In future work, we plan to compare models under the following assumptions: (1) participant-level cue weighting comes from a unimodal Gaussian distribution, (2) participant-level cue weighting comes from a bimodal distribution such that a participant either has cue weighting 1 or cue weighting > 1, and (3) participant-level cue weighting is constant, that is, 1 for all participants. If models (1) and (2) are better than (3), then there are individual differences in cue weighting. If model (2) is better than model (1), then cue-weighting variation among participants is split into two groups: participants with cue weighting 1 and participants with higher cue weighting.

Regarding our choice of parameters to fit, there are alternative choices available, which may result in different conclusions. While the choice of cue weighting is based on claims in the literature, the choice of the latency factor as representing reading speed and, by extension, language experience and fluency, may be somewhat more contentious. The latency factor is one of the most frequently estimated parameters in the ACT-R literature (Wong et al., 2010), but it is usually used to account for differences between studies rather than differences between individuals (Anderson et al., 1998). Other candidate sources of individual differences in ACT-R and the LV05 model are the activation noise parameter, the goal activation parameter, or the default action time parameter, which have been used in work on aphasia (Mätzig et al., 2018; Patil et al., 2016).

Additionally, reading speed as a global parameter, as indexed by the latency factor, is a multifaceted concept that can be measured with different tasks, only a subset of which may correlate with reading comprehension (Gerst et al., 2021; Jackson & McClelland, 1979). Ideally, in order to test the proposed connection between reading speed and cue weighting, participants’ reading speed should be measured independently, in a separate task. Another possibility is to compute average reading speed for filler sentences (Traxler et al., 2012), which we plan to do in future work.

We have assumed a simple relationship between reading speed and language skill: higher speed should be associated with more thorough syntactic processing. However, the data on this relationship are not as straightforward as one might hope. For instance, Roberts and Felser (2011) found lower comprehension accuracy for the faster readers in their native-speaker group for some garden-path sentences. Kaan et al. (2015) observed faster average reading in English for Dutch L2 learners than for English native speakers, as well as weaker online sensitivity to agreement errors for fast readers, both for native speakers and L2 learners. Findings like these suggest that reading speed does not necessarily correlate positively with experience or with comprehension. Readers may trade in accuracy for speed during reading, in accordance with whether their goal is detailed, holistic comprehension or something else, such as skimming the text for a particular type of information (Rayner et al., 2016). Reading may also become faster when memory retrievals fail due to lower working memory capacity (Nicenboim et al., 2016) or when certain aspects of the structural and semantic representation of the sentence are strategically left underspecified (Swets et al., 2008; von der Malsburg & Vasishth, 2013). Even if there is a stable underlying relationship between language experience, reading speed and comprehension, it may thus be obscured by varying task demands and differences in intrinsic and experiment-specific motivation (Schiefele et al., 2012). This is a challenge for almost all implemented models of sentence comprehension: Only a few implemented models (Logačev & Vasishth, 2015, 2016) explicitly address task effects on processing. Future work should investigate ways of including task effects in the LV05 model.

It is also not clear whether each individual has characteristic, fixed values for cue weighting and latency factor *within* a single study, as we have assumed here. This question can be answered by evaluating test-retest reliability, that is, by running the same experiment twice with each participant and checking whether the latency factor and cue weighting estimates from the first and second studies correlate. The reliability of individual differences in reading measures has recently started receiving more attention, as it is often unclear whether the observed differences between participants represent stable individual characteristics. While global reading speed has relatively high reliability, low reliability has been reported for the participant-level effects of some linguistic manipulations (Cunnings & Fujita, 2020; James et al., 2018; Staub, 2021), casting doubt on the assumption that individual differences can reliably be estimated in standard psycholinguistic experiments. Addressing this issue in the context of computational modeling is a further challenge. An important achievement of the present work is that we make these challenges explicit by using a computationally implemented model. Such a computational approach makes hypotheses more constrained and falsifiable than with a merely verbal model.

## CONCLUSION

Within sentence processing research, this work is, to our knowledge, the first attempt at simultaneously estimating multiple individual-level parameters and their correlation using approximate Bayesian computation. We have presented a novel investigation of the cue-weighting hypothesis and its implications for dependency completion in sentence comprehension. The theoretical insight from our modeling approach is that there is variation among individual speakers in the application of linguistic constraints, and that psycholinguistic hypotheses should be evaluated with regard to whether they hold for all, some, or none of the sampled participants. Furthermore, there is some indication that cue weighting may be tied to language experience, as reflected in average reading speed. While there are still many open questions, the computational and statistical approach pioneered by Turner and Van Zandt (2014) and others that we have applied here is broadly applicable across cognitive science; it can be easily adapted to different computational modeling settings.

## ACKNOWLEDGMENTS

The modeling work would not have been possible without the raw data provided by Brian W. Dillon, Sol Lago, and Matt Wagers; our heartfelt thanks to them. We also thank two anonymous reviewers for insightful comments.

## FUNDING INFORMATION

HY, Deutscher Akademischer Austauschdienst (https://dx.doi.org/10.13039/501100001655), Award ID: 91730718. SV, Deutsche Forschungsgemeinschaft (DFG, German Research Foundation), Project ID: 317633480, SFB 1287.

## AUTHOR CONTRIBUTIONS

HY: Conceptualization: Equal; Formal analysis: Lead; Methodology: Lead; Visualization: Lead; Writing - Original Draft: Equal; Writing - Review & Editing: Equal. DP: Conceptualization: Equal; Writing - Original Draft: Equal; Writing - Review & Editing: Equal. GS: Conceptualization: Equal; Formal analysis: Supporting; Methodology: Supporting; Supervision: Supporting; Writing - Original Draft: Equal; Writing - Review & Editing: Equal. BWD: Data Curation: Lead; Resources: Supporting; Writing - Review & Editing: Equal. SV: Conceptualization: Equal; Formal analysis: Supporting; Methodology: Supporting; Supervision: Lead; Writing - Original Draft: Equal; Writing - Review & Editing: Equal.

## Notes

^1^ SUBJ is an abstract proxy feature that stands in for all kinds of information that may help identify subjects, including but not limited to a structural dominance relationship with the verb (c-command), case information, and linear order.^2^ Other explanations for the observed facilitatory interference effect have been proposed in the literature; in this article, we only investigate the implications of the cue-based retrieval account.^3^ There are some complications associated with treating c-command as a retrieval cue: c-command is a relation between two nodes in a tree, which must be determined dynamically as the syntactic tree is incrementally built. This issue is generally circumvented by the simplifying assumption that the antecedent is in the subject position of the sentence. See the discussion in Dillon et al. (2013).^4^ These estimates were extracted from a Bayesian hierarchical linear model fit to the data; the individual estimates, shown here with 80% and 95% Bayesian credible intervals, are so-called shrunken estimates for each participant. Such shrunken estimates are informed by the grand mean, and are more conservative than the estimates computed by estimating each individual’s mean effect in isolation (Bates et al., 2014).^5^ We use the term reading speed informally to refer to reading latency.^6^ Reading speed refers to an individual’s overall reading fluency, which is assumed to be constant throughout the experimental trials. Since we do not have any independent measure of reading speed, as a first approximation we use reading times to estimate the reading speed parameter for an individual. We discuss this later in detail.^7^ In ACT-R, a retrieval threshold parameter determines whether the activation is high enough for successful retrieval; when the activation falls below this threshold, retrieval failure occurs. This is how occasional retrieval failures are modeled in ACT-R.^8^ Given our prior specifications for the cue weighting parameter, a large cue weighting here is approximately equal to 4.^9^ This is a simplifying assumption, as reading speed may be influenced by a multitude of factors. We will discuss alternative approaches in the general discussion.

## Figures and Tables

**Figure A1.**
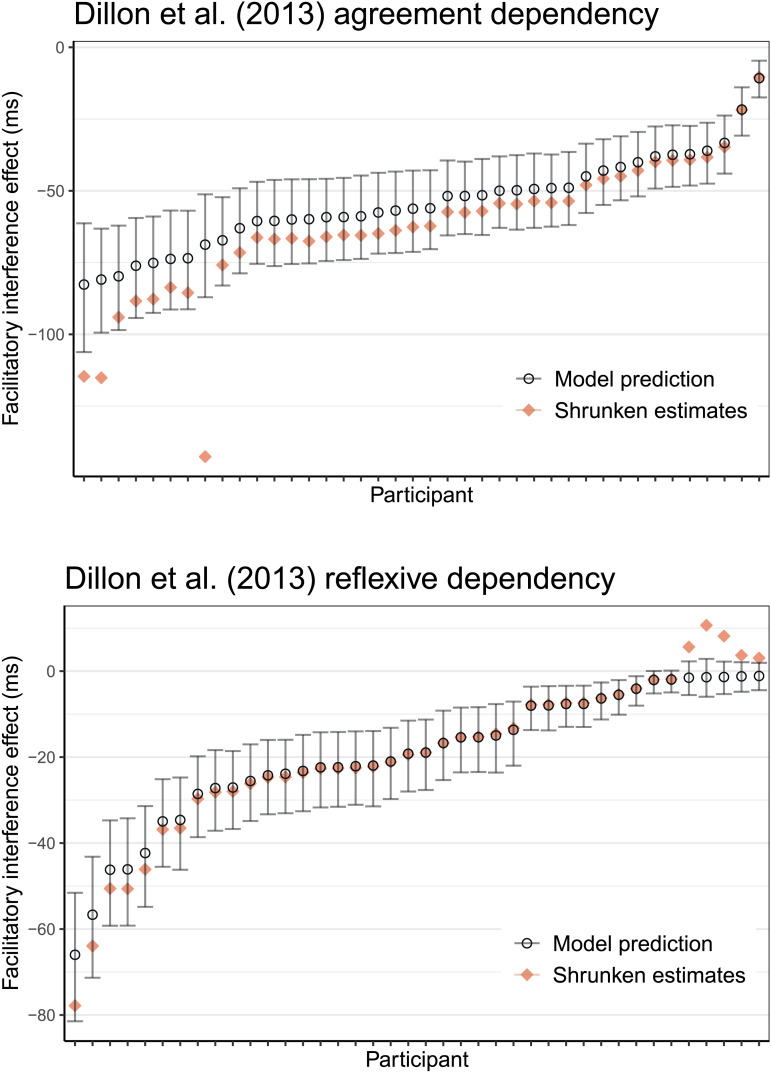
**Posterior predicted values for individuals in agreement and reflexive dependencies, derived from the cue-based retrieval model after estimating individual-level parameters for cue weighting.** Shown are the posterior mean and 95% credible interval, along with the estimate of the shrunken mean for each participant.

## Supplemental Materials

https://osf.io/3na9q/
