# Supplementary material for: Individual Differences in Cue Weighting in Sentence Comprehension: An Evaluation Using Approximate Bayesian Computation
Source: Open Mind (Camb). 2022 Jul 1;6:1–24. doi: 10.1162/opmi_a_00052 (PMC9692063; doi:10.1162/opmi_a_00052)
Supplement: Supplementary file 1 [file opmi-06-1-s001.pdf]

Supplementary Materials

Individual differences in cue weighting in sentence comprehension: An evaluation using Approximate Bayesian Computation

Himanshu Yadav, Dario Paape, Garrett Smith, Brian W. Dillon, and Shravan Vasishth

Contents

S1 Hierarchical ABC algorithm **1**
S1.1 Hierarchical model and the posterior distributions 1
S1.2 Hierarchical ABC algorithm 4
S1.3 Validation 5

S2 Random effects meta-analysis of correlations **8**

References **9**

S1 Hierarchical ABC algorithm

S1.1 Hierarchical model and the posterior distributions

Suppose that y_j are the data (interference effect) associated with the jth participant, LF_j and CW_j are the latency factor and the cue weighting parameters respectively for the jth participant.

$$y_j \sim Model(\theta_j) \tag{1}$$

$$\text{Where, } \theta_j = \begin{pmatrix} LF_j \\ CW_j \end{pmatrix}$$

We assume that LF_j and CW_j come from a multivariate normal distribution with population means μ_{LF} and μ_{CW}, population standard deviations σ_{LF} and σ_{CW}, and correlation parameter, ρ.

$$\begin{pmatrix} LF_j \\ CW_j \end{pmatrix} \sim \mathcal{N}_2\left(\mu = \begin{pmatrix} \mu_{LF} \\ \mu_{CW} \end{pmatrix}, \Sigma = \begin{pmatrix} \sigma^2_{LF} & \rho\sigma_{LF}\sigma_{CW} \\ \rho\sigma_{LF}\sigma_{CW} & \sigma^2_{CW} \end{pmatrix}\right) \tag{2}$$

Priors

The prior distributions over the parameters are plotted in Figure S1.

$$\begin{pmatrix}\mu_{LF}\\ \mu_{CW}\end{pmatrix} \sim \mathcal{N}_2\left(\begin{pmatrix}\mu_{0,LF}\\ \mu_{0,CW}\end{pmatrix}, \Lambda_0\right)$$

$$\text{where } \mu_{0,LF} = 0.15,\ \mu_{0,CW} = 2.5, \text{ and } \Lambda_0 = \begin{pmatrix}.03^2 & 0\\ 0 & 10^2\end{pmatrix}$$

$$\Sigma \sim InverseWishart\left(\nu = 5, \Psi = \begin{pmatrix}0.1 & 0\\ 0 & 0.1\end{pmatrix}\right)$$

Figure S1

Prior distributions for the population-level parameters: population mean latency factor, population mean cue weighting, population standard deviation latency factor, population standard deviation cue weighting, and the correlation parameter.

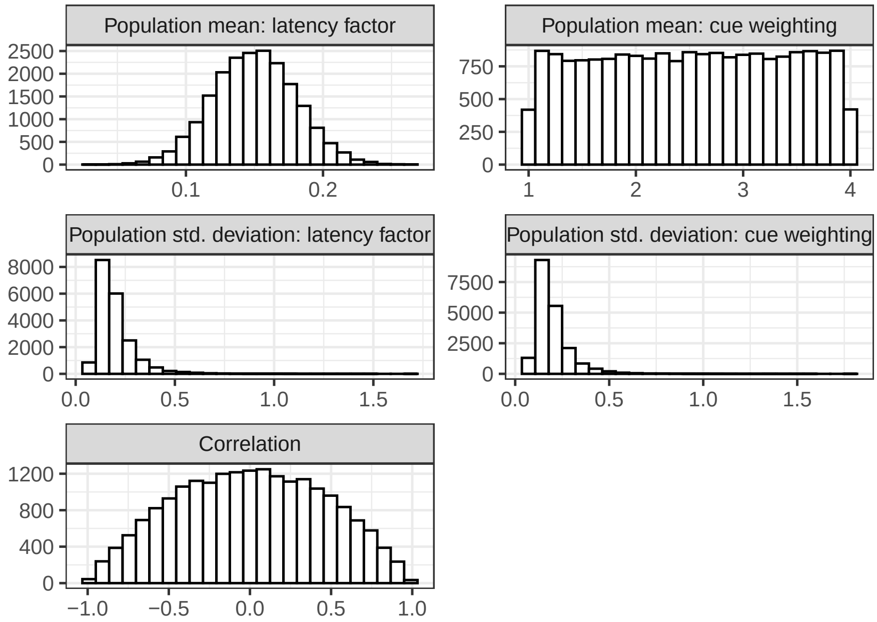

The rationale for choosing the above priors is that they are conjugate priors for a multivariate normal distribution. Given these priors, we can write full conditional posterior distribution for population level parameters, μ and Σ conditional on individual-level parameters $\{\theta_1, \theta_2, .., \theta_n\}$. Full conditional distributions will be useful for sampling, as we use Gibbs sampling to sample population-level parameters. We describe the posterior distributions of individual-level and population-level parameters next.

Posterior distributions

Consider posterior distribution for the individual-level parameters $\{\theta_1, \theta_2, .., \theta_n\}$ conditional on data y and population-level parameters μ and Σ, where $\mu = \begin{pmatrix} \mu_{LF} \\ \mu_{CW} \end{pmatrix}$, and $\Sigma = \begin{pmatrix} \sigma^2_{LF} & \rho\sigma_{LF}\sigma_{CW} \\ \rho\sigma_{LF}\sigma_{CW} & \sigma^2_{CW} \end{pmatrix}$.

$$\pi(\theta_1, .., \theta_n | y, \mu, \Sigma) \propto \pi(y|\theta_1, .., \theta_n, \mu, \Sigma)\pi(\theta_1, .., \theta_n|\mu, \Sigma) \tag{3}$$

Given that individual-level parameters θ_js are independent, we can write joint posterior density of $\{\theta_1, \theta_2, .., \theta_n\}$ as product of posterior density of individual θ_j,

$$\pi(\theta_1, .., \theta_n | y, \mu, \Sigma) \propto \prod_{j=1}^{n} \pi(y_j|\theta_j)\pi(\theta_j|\mu, \Sigma) \tag{4}$$

We know that μ and Σ have no role in the probability density of the data y_j, and y_j depends on $\{\mu, \Sigma\}$ only through θ_j. The conditional distribution of each of the θ_j depends on the the data exclusive to the jth subject,

$$\pi(\theta_j | y_j, \mu, \Sigma) \propto \pi(y_j|\theta_j)\pi(\theta_j|\mu, \Sigma) \tag{5}$$

ABC method allows us to approximate the conditional posterior for individual-level parameters θ_j as

$$\pi(\theta_j | y_j, \mu, \Sigma) \propto \Psi(dist(S(y_j), S(x_j))|\delta)\pi(\theta_j|\mu, \Sigma) \tag{6}$$

Where $\Psi(\cdot|\delta)$ is a Kernel function, x_j is simulated data from the model for θ_j, and S($\cdot$) is a summary statistic.

Now consider the posterior distribution for Σ conditional on individual level parameters $\theta_{1:n}$ and population-mean $\mu = \begin{pmatrix} \mu_{LF} \\ \mu_{CW} \end{pmatrix}$,

$$\Sigma|\theta_{1:n}, \mu \sim InverseWishart(\Psi_n, \nu_n) \tag{7}$$

Where,

$$\Psi_n = \Psi + \sum_{j=1}^{n}(\theta_j - \bar{\theta})(\theta_j - \bar{\theta})^T + n(\bar{\theta} - \mu)(\bar{\theta} - \mu)^T$$

$$\nu_n = \nu + n$$

$$\bar{\theta} = \textstyle\sum_{j=1}^{n} \theta_j$$

And, posterior distributions for $\mu = \begin{pmatrix} \mu_{LF} \\ \mu_{CW} \end{pmatrix}$ conditional on Σ and $\theta_{1:n}$ will be a bivariate normal distribution,

$$\mu|\theta_{1:n}, \Sigma = \mathcal{N}_2\left(\begin{pmatrix} \mu_{LF_n} \\ \mu_{CW_n} \end{pmatrix}, \Sigma_n\right) \tag{8}$$

Where,

$$\begin{pmatrix} \mu_{LF_n} \\ \mu_{CW_n} \end{pmatrix} = (\Lambda_0^{-1} + n\Sigma^{-1})^{-1}(\Lambda_0^{-1}\mu_0 + n\Sigma^{-1}\bar{\theta})$$

with $\mu_0 = \begin{pmatrix} \mu_{0,LF} \\ \mu_{0,CW} \end{pmatrix}$ and $\Sigma_n^{-1} = \Lambda_0^{-1} + n\Sigma^{-1}$

S1.2 Hierarchical ABC algorithm

In order to estimate both participant-level and population-level parameters, we use a hierarchical ABC algorithm which samples from the posterior distributions stated in the previous section. We use the Gibbs ABC algorithm proposed by Turner and Van Zandt (2014). In the Gibbs ABC algorithm, on each iteration, the participant-level parameters are sampled from the approximate posterior distribution using an ABC method, and then each population-level parameter is sampled from a posterior distribution conditional on all other population- and participant-level parameters. We have derived the conditional posterior distributions for all the parameters in the previous section. A hierarchical ABC algorithm works in the following steps. First, initialize all population-level and participant-level parameters, i.e., μ, Σ and $\theta_{1:n}$. After that, in each iteration:

(1) Sample participant-level parameters, i.e., θ_i using ABC method from the approximate conditional posterior distribution shown in equation 6.

(2) Sample population-level variance-covariance matrix, Σ from the posterior distribution conditional on current state of participant-level parameters and population means as shown in equation 7.

(3) Sample population-level means, μ from the posterior distribution conditional on participant-level parameters, $\theta_{1:n}$ and population-level variance Σ as shown in equation 8.

As discussed earlier, ABC algorithms have their internal parameters i.e., the tolerance parameter δ and the choice of summary statistics $S(\cdot)$. As δ approaches 0 and if the summary statistic is sufficient[1], the approximation of the desired posterior distribution becomes exact. If δ is too low, though, sampling becomes difficult. Therefore, a reasonable value of δ has to be chosen such that it makes a good approximation in the given time. Here, we choose δ in the range [0.1, 0.2], with the value of δ in our algorithm decreasing in each iteration. As we show in the validation section, the above range of δ makes a good approximation in a reasonable amount of time. We choose the *mean* of the data as the summary statistic for calculating the discrepancy between observed data and model-generated data.

[1]A summary statistic, say S, is sufficient for a sample if the sample gives no additional information than the statistic S

Algorithm S1

Hierarchical Gibbs ABC algorithm for individual-level parameter estimation

1	At iteration t=1
	Initialize a pool of K samples for μ, Σ, and $\theta_{1:n} = \theta_1, ..\theta_n$
	Set equal weights for each sample of $\theta_{1:n}$ as $W_{1:n,1,1}, ..W_{1:n,1,2}, ..W_{1:n,1,K} = 1/K$
2	**for iteration** $2 \leq t \leq T$
3	**for sample** $2 \leq k \leq K$
4	Sample $\mu_{t,k}$ from conditional posterior $\pi(\mu \mid \theta_{1:n,t,k-1}, \Sigma_{t,k-1})$
5	Sample $\Sigma_{t,k}$ from conditional posterior $\pi(\Sigma \mid \theta_{1:n,t,k-1}, \mu_{t,k})$
6	**for participant** $1 \leq j \leq n$
7	Sample a value θ_j^* from $\theta_{j,t-1,1:K}$ with probabilities $W_{j,t-1,1:K}$
8	Perturb θ_j^* by sampling $\theta_j^{**} \sim Normal(\theta^*, \sigma)$
9	Generate values from the model for θ_j^{**} : $x_j \sim Model(\theta_j^{**})$
10	Calculate weight for θ_j^{**} as $\Psi(dist(S(x_j), S(Y_j)) \mid \delta_t) * \pi(\theta_j^{**} \mid \mu, \Sigma)$
11	**end for**
12	**end for**
13	**end for**

S1.3 Validation

We validate the performance of the hierarchical ABC algorithm using simulated data from the model. We simulate participant-level facilitatory interference effect data from 1 for 50 fake participants using a set of parameters values described in the table S2. Then, we compute the participant level estimates as well as population level estimates for latency factor and cue weighting using the Hierarchical ABC algorithm. The comparison of true parameter values and estimated parameters values is shown in figures S2 and S3.

Table S2

Set of true parameter values for the population-level parameters

**Parameter**		**True value**
Population mean: Latency factor	μ_{LF}	0.5
Population mean: Cue weighting	μ_{CW}	2.0
Population variance: Latency factor	σ^2_{LF}	0.02
Population variance: Cue weighting	σ^2_{CW}	0.08
Correlation	ρ	-0.5

Figure S2

Estimated posterior distributions for the population-level parameters; the corresponding black vertical lines represent true parameter values.

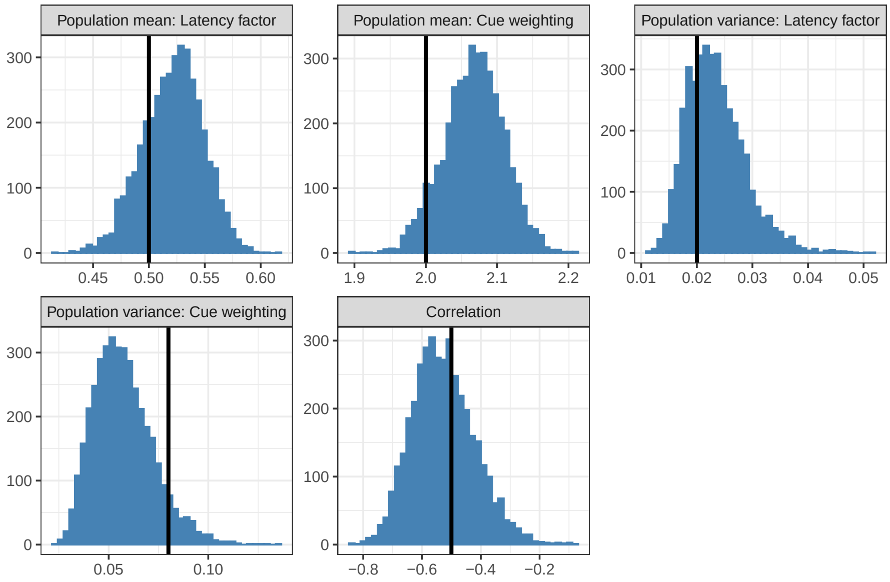

Figure S3

Estimated posterior distributions for latency factor and cue weighting for each participant are shown as blue histograms and true parameter values are shown as black vertical lines.

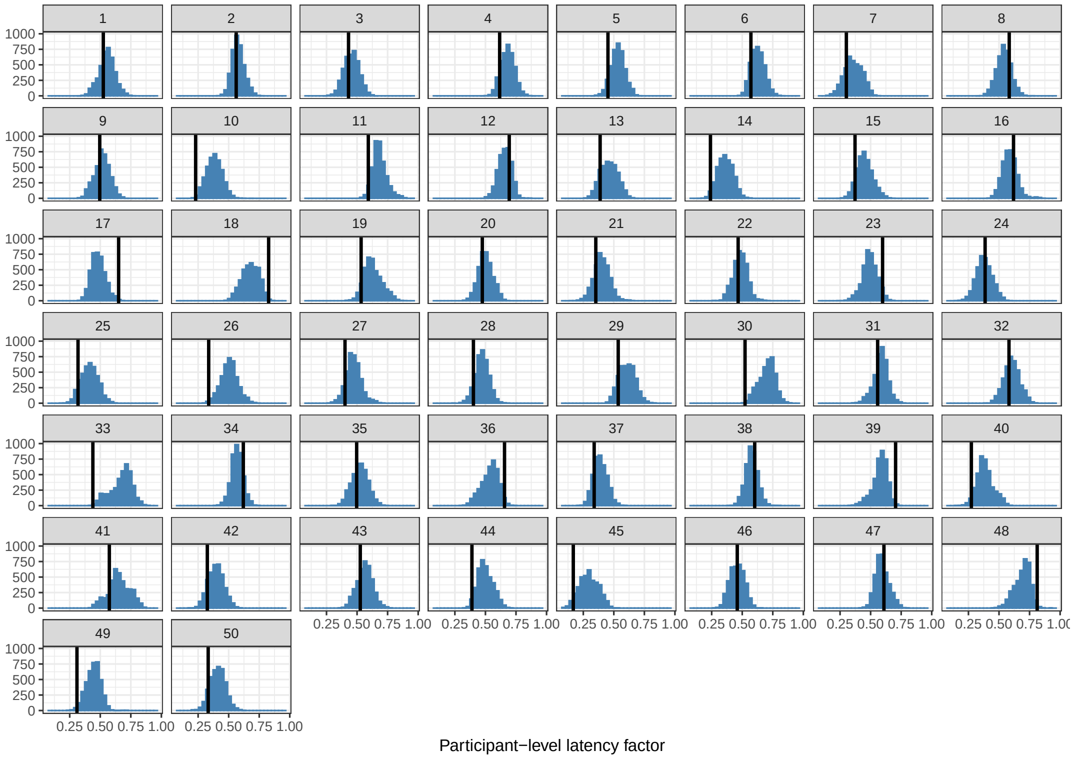

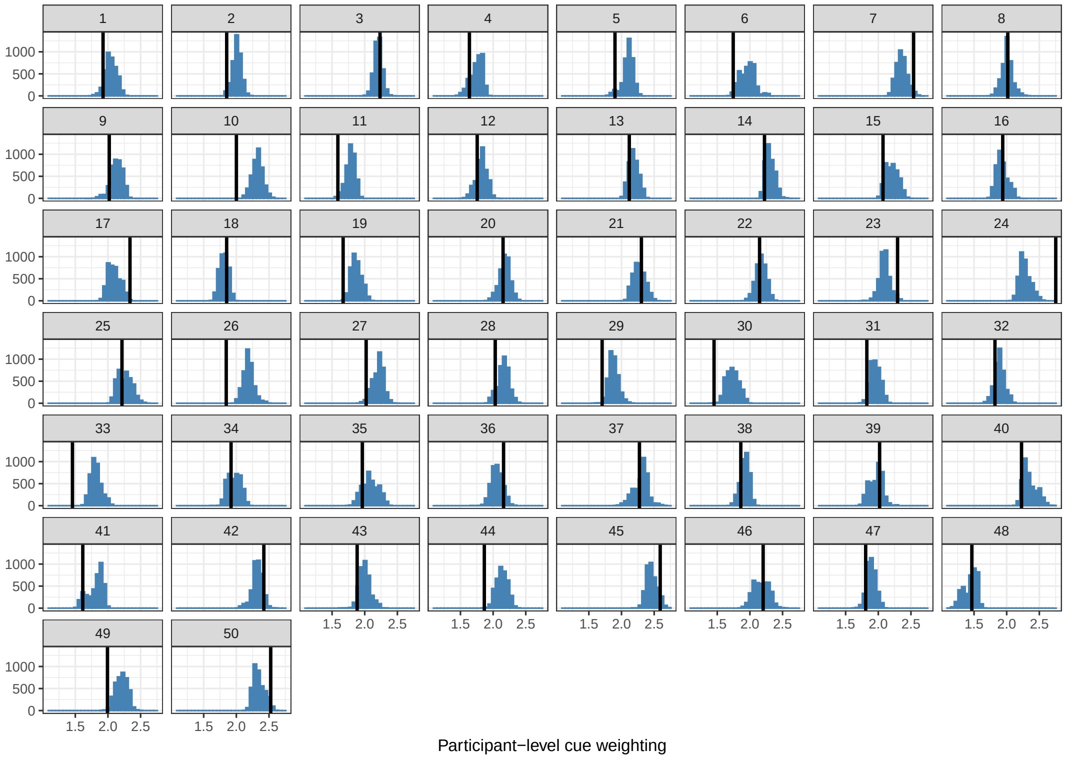

S2 Random effects meta-analysis of correlations

For a dataset i which had n independent participants, suppose the estimated correlation between latency factor and cue weighting is r_i. The Fisher z-transformation of correlation r_i is given as

$$z_i = \frac{1}{2} \ln \frac{1 + r_i}{1 - r_i}$$

$z_1, z_2, ...,$ approximately follow a normal distribution with mean $\frac{1}{2} \ln \frac{1+\rho}{1-\rho}$ and variance $\phi = \frac{1}{n-3}$ (Fisher, 1921):

$$z_i \sim Normal(\frac{1}{2} \ln \frac{1 + \rho}{1 - \rho}, \frac{1}{n - 3})$$

where ρ is the population-level correlation between latency factor and cue weighting, and n is the number of participants in the ith dataset.

In order to implement this, we first derive the Fisher z-transformation of correlation estimates from all 13 datasets, $z_1, z_2, ..., z_{13}$. We then calculate variance, $\phi_i = \frac{1}{n-3}$ from number of participants n for each dataset. We fit a random-effects model such that Fisher z-transformation of correlation estimate for i^{th} dataset come from a normal distribution with mean μ_i and variance ϕ_i:

$$z_i \sim Normal(\mu_i, \phi_i)$$

and

$$\mu_i \sim Normal(z_p, \sigma^2)$$

where z_p is Fisher z-transformation of population-level correlation ρ i.e., $z_p = \frac{1}{2} \ln \frac{1+\rho}{1-\rho}$.

The priors on the parameter are: $z_p \sim Normal(0, 1)$ and $\sigma \sim Normal_+(0, 1)$; the prior on σ is truncated at 0.

We fit the above model using the brms package (Bürkner, 2017) and estimated the parameters z_p and σ. The parameter z_p is transformed back to the original scale to obtain the population-level correlation between latency factor and cue weighting.

$$\rho = \frac{exp(2z_p) - 1}{exp(2z_p) + 1}$$

References

Bürkner, P.-C. (2017). brms: An R package for Bayesian multilevel models using Stan. *Journal of Statistical Software*, *80*(1), 1–28.

Fisher, R. A. (1921). On the 'probable error' of a coefficient of correlation deduced from a small sample. *Metron*, *1*, 1–32.

Turner, B. M., & Van Zandt, T. (2014). Hierarchical approximate Bayesian computation. *Psychometrika*, *79*(2), 185–209.
